# Prevalence and Incidence of Attention Deficit/Hyperactivity Disorder in Denmark. A National Register‐Based Open Cohort Study

**DOI:** 10.1111/acps.13804

**Published:** 2025-03-27

**Authors:** Simon Grøntved, Kathrine Hald, Christina Mohr‐Jensen, Søren Paaske Johnsen, Jan Mainz, Jan Brink Valentin

**Affiliations:** ^1^ Psychiatry The North Denmark Region Aalborg Denmark; ^2^ Danish Center for Health Services Research, Department of Clinical Medicine Aalborg University Aalborg Denmark; ^3^ Medical Diagnostic Centre, University Clinic for Development of Innovative Patient Pathways Silkeborg Regional Hospital Silkeborg Denmark; ^4^ Institute of Communication and Psychology, Psychology Aalborg University Aalborg Denmark

**Keywords:** ADHD, attention deficit/hyperactivity disorder, incidence, prevalence

## Abstract

**Introduction:**

Rises in prevalence and incidence of the neurodevelopmental disorder Attention‐deficit/Hyperactivity Disorder (ADHD) have been reported globally and estimated in Denmark previously. Nevertheless, differences in methodology hinder an assessment of the temporal evolution of ADHD based on published literature. The aim of this study was to consistently calculate the yearly prevalence and incidence proportions of ADHD and the use of ADHD medication in Denmark from 2000 to 2022.

**Methods:**

We conducted an open cohort study using register data covering the entire Danish population. We defined ADHD as either having a hospital diagnosis of ADHD (ICD‐10: F90.0, F90.1, F90.8, or F98.8), or having redeemed a prescription for ADHD medication.

**Results:**

Of the included 7,748,837 persons, 2.52% had ADHD, of whom 58.33% were males. The prevalence of ADHD has increased from 0.10% in 2000 to 3.03% in 2022. Specifically for the 18–27‐year‐olds, the prevalence was 8.16% for males and 6.12% for females in the year 2022. For the incidence, the same age group peaked within the females in 2022 at 1.10%, whereas the male incidence was consistently highest in the 6–18‐year‐olds, peaking in 2022 at 0.94%. Total use of ADHD medication in the population also increased. Methylphenidate was used by 97.58% of the ADHD medicated patients in 2000 and 70.60% in 2022. Lisdexamphetamine was the second most common in 2022, where 24.03% redeemed a prescription.

**Limitations:**

Incidence and prevalence were calculated specifically for the Danish population and may not be directly generalizable to other countries.

**Conclusions:**

The prevalence of ADHD has consistently increased throughout the past two decades. The main contribution to this increase is the ADHD incidence in females, especially young females in the most recent years 2020–2022. Methylphenidate was consistently the most common ADHD drug prescribed, though lisdexamphetamine use has increased in recent years.

**Summary**

- Significant outcomes
  ○ Prevalence and incidence of diagnosed attention deficit/hyperactivity disorder (ADHD) have increased notably over the past two decades. The prevalence of ADHD in males aged 18 to 27 years reached 8.16% in the year 2022.
  ○ The incidence of ADHD in young females increased significantly from 2019 to 2022.
  ○ In 2000 almost all medicated ADHD patients were prescribed methylphenidate, and it remained the most common choice of drug in 2022, where it was prescribed to 70.60% of the patients. In 2022, lisdexamphetamine was prescribed to 24.03%, and atomoxetine to 19.32% of the patients.
- Limitations
  ○ Prevalence and incidence were calculated specifically for the Danish population and may not be directly generalizable to other countries.
  ○ Patients diagnosed by private practicing psychiatrists who had not redeemed a prescription for ADHD medication were not included in the analyses. Thus, the prevalence and incidence estimates were likely underestimated in this study.

## 1 Introduction

### 1.1 Background

Attention‐deficit/Hyperactivity Disorder (ADHD) is a neurodevelopmental disorder characterized by symptoms of inattention, hyperactivity, impulsiveness, and associated functional impairment [1]. ADHD is associated with numerous challenges across the lifespan. These include academic difficulties, reduced educational and occupational outcomes, fewer lifetime opportunities due to lower wages and occupational rank, social relationship difficulties, and engagement in high‐risk behavior, such as substance abuse, higher risk of accidents, lower health‐related quality of life, and lower self‐esteem [2, 3, 4]. Moreover, ADHD is linked to somatic and mental health issues such as cardiovascular disease and depression, which affect quality of life as well as being associated with increased healthcare costs [5, 6, 7]. The negative impact of ADHD further extends to relatives and caregivers. Young adults with ADHD may also face financial dependence on family members and reliance on welfare systems [8, 9, 10, 11].

Estimates of the prevalence and incidence of any diagnosis vary as a consequence of methodological approach. The ADHD prevalence was estimated in a meta‐analysis to be 1.1% worldwide in 2019, and 2.7% specifically among children and adolescents [12, 13]. However, other systematic reviews have found a markedly higher prevalence of 3.1% in adults, and of 5.4%–8.1% in children and adolescents [13, 14, 15]. Estimates from these meta‐analyses likely differ as a consequence of geographical variation as well as differences in their method of data aggregation. Furthermore, there were remarkable differences between the reported prevalences and incidences of the reviewed studies included in the meta‐analyses, especially the difference between studies that screened a population for symptoms and studies that relied on registered diagnoses. For instance, Rowland et al. reported a prevalence of 15.5% in 2015 in American school children based on screening interviews conducted with parents and teachers [16]. In comparison, Polyzol et al. [17] estimated the ADHD prevalence to be 0.4% in the adult Swedish population in 2011 based on diagnoses of ADHD from national health registers. Similarly, Holden et al. [18] used a database from primary care physicians to estimate the overall prevalence of ADHD in the British population to be 0.1% in 2011.

Given the wide‐ranging risks associated with ADHD, policymakers and healthcare planners must monitor prevalence trends and align them with the best available knowledge to ensure resources are effectively allocated. Care pathways are needed in order to provide high‐quality support, improving outcomes for individuals and reducing societal costs [19, 20, 21, 22, 23]. To estimate the temporal change in ADHD prevalence and incidence in Denmark, there is a need for a consistent calculation of yearly prevalence and incidence.

### 1.2 Objectives

The aim of this study was to examine the prevalence and incidence of ADHD diagnosis and the use of ADHD medication in Denmark from 2000 to 2022.

## 2 Methods

The study was conducted in accordance with the STrengthening the Reporting of OBservational studies in Epidemiology (STROBE) guideline for cohort studies [24].

### 2.1 Study Design

We conducted an open cohort study using Danish register data covering the entire Danish population.

### 2.2 Setting

The Danish population consists of approximately 6 million people living in a high‐income society with a publicly funded, universal health care system. The health care system covers all citizens and aims at equal access with high‐quality care for all citizens [25].

### 2.3 Data Sources

In the highly digitalized Danish administration system, registers can be unambiguously linked on an individual level using the personal identification number given to all Danish residents upon birth or immigration. We linked information from the following registers: *The National Patient Register* is a nationwide registry containing data on all hospital admissions, outpatient visits, and emergency room visits in Denmark since 1977 [26]. *The Danish National Prescription Registry* records all redeemed prescriptions in Danish pharmacies since 1995, providing data on drug use at the individual level [27]. *The Danish Civil Registration Register*, established in 1968, tracks all residents in Denmark, including their vital status, family relations, and migration history [28]. *The Register of Causes of Death* provides nationwide data on causes of death in Denmark, coded according to the International Classification of Diseases (ICD) [29].

### 2.4 Population

All Danish residents between 1 January 2000 to 31 December 2022 were included in the study. Data for classification of ADHD was available from 1994, which gave us a lead‐in period of six years to reduce underestimation of prevalence and overestimation of incidence. The subjects were followed from the start of the study period, birth or immigration to the end of the study period, death or emigration. Death or emigration excluded a person from all subsequent years until immigration if such occurred. Thus, subjects could be included multiple times in the cohort if emigration was not permanent throughout the study period.

### 2.5 Outcomes

In the study, a person was classified as having ADHD if the person had a registered *hospital diagnosis of ADHD* or had redeemed a prescription for *ADHD medication*. Persons not meeting these criteria in a given year were considered to be living without ADHD. As a consequence of this definition, a person could not revert from having ADHD.

*Hospital diagnosis* of ADHD was defined using ICD10 categories F90.0, F90.1, F90.8, and F98.8. Any contact with a Danish hospital, resulting in a primary or secondary diagnosis of ADHD, categorized the person as having ADHD from the start date of that contact until the end of the study period.

*ADHD medication* was defined as any prescription drug with the following active ingredients with associated Anatomical Therapeutic Chemical (ATC) codes: Guanfacine (C02AC02), dexamphetamine (N06BA02), methylphenidate (N06BA04), modafinil (N06BA07), atomoxetine (N06BA09), or lisdexamphetamine (N06BA12). Redeeming a prescription with one of these ingredients categorized a person as *medicated* with that drug. To minimize the risk of misclassification, we did not categorize a person as receiving treatment for ADHD if treated with modafinil and having a diagnosis of narcolepsy (ICD10:G47.4).

### 2.6 Covariates

The cohort was analyzed in strata of sex and age groups. Sex was defined using the sex (male/female) as assigned at birth. Age was calculated in whole years from birth year and categorized into seven groups. Two age groups for children who are seen in child and adolescent psychiatry services: 0–6 preschool and 6–18 primary education. Adults were grouped in five age groups: 18–27 professional education, 27–35 early career, 35–50 middle career, 50–70 late career, and 70+ retired [30, 31, 32, 33].

### 2.7 Statistical Methods

The prevalence and incidence proportions of ADHD in Denmark were calculated annually and presented as percentages. Yearly prevalence proportion was defined as the percentage of the population with ADHD prior to or within each year, while the incidence proportion was defined as the percentage of the undiagnosed population who were diagnosed for the first time within each year. Both metrics were stratified by age and sex, with population numbers by stratum shown in Figure S4. We further calculated the male/female ratio of prevalence and incidence proportions, along with their 95% confidence intervals.

The use of ADHD medication was analyzed by reporting the percentage of the population who redeemed prescriptions for any form of ADHD medication each year, stratified by sex and age. Additionally, the proportion of the medicated population who redeemed prescriptions for medication with each specific active ingredient was calculated.

All results were presented as graphs with underlying data provided in supplementary tables for transparency. Proportions were presented as percentages with 95% confidence intervals. Missing diagnoses or prescriptions were treated as absent, with minimal impact expected due to the recurring nature of the data. As a supplementary analysis, the prevalence and incidence proportions were also estimated based exclusively on diagnoses. Analyses were conducted in R version 4.1.1.

## 3 Results

### 3.1 Population

We identified 7,748,837 Danish residents from the Danish Central Person Register whom we followed between 2000 and 2022 for 128,006,981 person‐years combined. Table 1 shows the numeric size of the population and person time for each individual year.

**TABLE 1:** The cumulated number of person years calculated in days, rounded to whole years, stratified by sex and year. Number of unique individuals living in Denmark at some point within a given year, stratified by sex. Number of individuals prevalent, incident, and medicated each year, stratified by sex.

Unique individuals and person time															
	Person years			Individuals living in Denmark			Subpopulation prevalent with ADHD			Subpopulation incident with ADHD			Subpopulation medicated for ADHD		
	Females	Males	All	Females	Males	All	Females	Males	All	Females	Males	All	Females	Males	All
2000	2,695,425	2,634,625	5,330,051	2,701,908	2,641,066	5,342,974	1523	3860	5383	244	794	1038	507	1517	2024
2001	2,697,027	2,636,660	5,333,687	2,710,985	2,650,488	5,361,473	1792	4693	6485	303	875	1178	568	1756	2324
2002	2,703,918	2,644,987	5,348,906	2,717,816	2,658,819	5,376,635	2098	5760	7858	360	1110	1470	708	2223	2931
2003	2,710,058	2,652,315	5,362,373	2,723,958	2,666,036	5,389,994	2536	7081	9617	494	1395	1889	933	2829	3762
2004	2,724,227	2,666,551	5,390,778	2,730,755	2,673,444	5,404,199	3260	8786	12,046	788	1783	2571	1353	3711	5064
2005	2,723,937	2,667,435	5,391,373	2,738,270	2,681,835	5,420,105	4146	10,814	14,960	954	2118	3072	1848	4879	6727
2006	2,732,854	2,678,444	5,411,298	2,747,977	2,693,818	5,441,795	5332	13,369	18,701	1302	2654	3956	2600	6523	9123
2007	2,744,384	2,693,134	5,437,519	2,759,759	2,708,852	5,468,611	7160	16,864	24,024	1935	3616	5551	3861	8806	12,667
2008	2,766,890	2,717,918	5,484,808	2,775,092	2,727,068	5,502,160	9886	21,825	31,711	2842	5118	7960	5766	12,605	18,371
2009	2,771,349	2,721,344	5,492,693	2,786,982	2,737,896	5,524,878	13,401	28,355	41,756	3653	6702	10,355	8305	17,419	25,724
2010	2,783,031	2,733,776	5,516,808	2,798,770	2,750,115	5,548,885	17,311	35,084	52,395	4075	6956	11,031	10,760	21,759	32,519
2011	2,792,970	2,744,707	5,537,677	2,808,805	2,761,012	5,569,817	21,114	40,903	62,017	3997	6093	10,090	12,505	23,885	36,390
2012	2,809,974	2,763,701	5,573,675	2,818,362	2,772,534	5,590,896	24,738	46,474	71,212	3835	5861	9696	13,873	25,254	39,127
2013	2,813,112	2,768,979	5,582,091	2829,345	2,785,958	5,615,303	28,235	51,055	79,290	3686	4870	8556	14,740	25,065	39,805
2014	2,827,295	2,788,040	5,615,336	2,843,736	2,805,263	5,648,999	31,583	55,669	87,252	3561	4945	8506	15,388	24,962	40,350
2015	2,846,818	2,813,379	5,660,198	2,863,872	2,831,289	5,695,161	35,010	60,308	95,318	3649	5015	8664	16,516	25,791	42,307
2016	2,873,048	2842,272	5,715,321	2,882,127	2,852,487	5,734,614	38,465	64,947	103,412	3777	4991	8768	17,460	26,735	44,195
2017	2,880,869	2,850,576	5,731,446	2,897,924	2,868,564	5766,488	42,332	70,121	112,453	4113	5559	9672	19,049	28,468	47,517
2018	2,893,717	2,863,190	5,756,907	2,910,596	2,881,258	5,791,854	46,212	75,323	121,535	4154	5619	9773	20,822	30,691	51,513
2019	2,905,069	2,874,256	5,779,325	2,921,368	2,891,605	5,812,973	50,462	81,299	131,761	4550	6339	10,889	22,640	33,507	56,147
2020	2,924,571	2,892,302	5,816,873	2,932,432	2,901,280	5,833,712	55,769	87,863	143,632	5547	6971	12,518	25,321	36,461	61,782
2021	2,933,376	2,903,864	5,837,241	2,948,555	2,920,166	5,868,721	63,495	95,651	159,146	8060	8236	16,296	30,938	41,234	72,172
2022	2,968,106	2,932,490	5,900,597	2,982,630	2,947,459	5,930,089	74,480	105,152	179,632	11,374	10,095	21,469	39,295	47,412	86,707
Total	64,522,025	63,484,945	128,006,981	3,873,839	3,874,998	7,748,837	81,454	114,018	195,472	81,454	114,018	195,472	73,687	101,590	175,277

### 3.2 Descriptive Data

Of the included 7,748,837 persons, 195,472(2.52%) were categorized with ADHD by our definition and lived in Denmark within at least one of the years between 2000 and 2022. Of these, the majority (58.33%) were male. Numbers for specific years, as well as stratified by sex, can also be seen in Table 1.

### 3.3 Prevalence and Incidence

**FIGURE 1:**
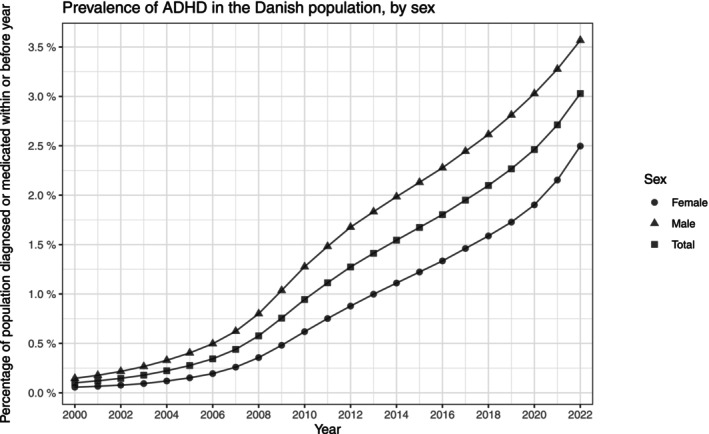
The prevalence proportion of Attention Deficit/Hyperactivity Disorder (ADHD) in Denmark between the years 2000 and 2022. The entire population stratified by sex.

**FIGURE 2:**
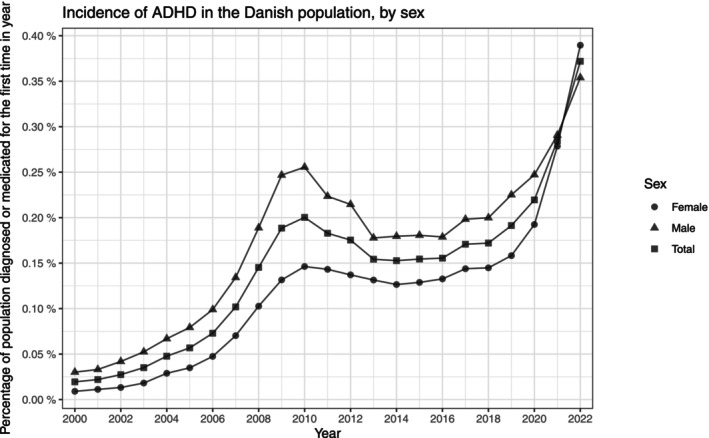
The incidence proportion of Attention Deficit/Hyperactivity Disorder (ADHD) in Denmark between the years 2000 and 2022. The entire population stratified by sex.

Both the prevalence and incidence proportions of ADHD increased throughout the entire study period. In 2000, the prevalence was 0.10% (0.10%, 0.10%) and increased to 3.03% (3.02%, 3.04%) in 2022. The incidence increased in the years up to 2010, while a temporary decline was observed in the years 2011–2013, followed by a stable period in 2014–2016. The incidence was 0.02% (0.02%, 0.02%) in 2000 and increased to 0.37% (0.37%, 0.38%) in 2022. The female incidence in 2022 surpassed that of males after having been consistently lower throughout the study period. The yearly prevalence was reported in Figure 1, and the incidence in Figure 2; the corresponding counts are in Table S1. The male/female ratio between the prevalences peaked at 2.85(2.73, 2.99) in 2003, since when it has decreased, reaching 1.43 (1.42, 1.44) in 2022 (Figure S5 and Table S1). Additionally, the male/female ratio for the incidences decreased consistently from 3.33 (2.89, 3.85) in 2000 to 0.91 (0.88, 0.93) in 2022.

**FIGURE 3:**
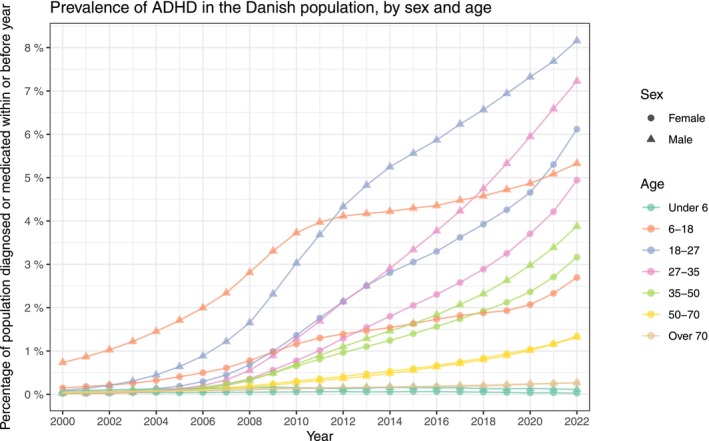
The prevalence proportion of Attention Deficit/Hyperactivity Disorder (ADHD) in Denmark between the years 2000 and 2022. The entire population stratified by sex and age‐group.

**FIGURE 4:**
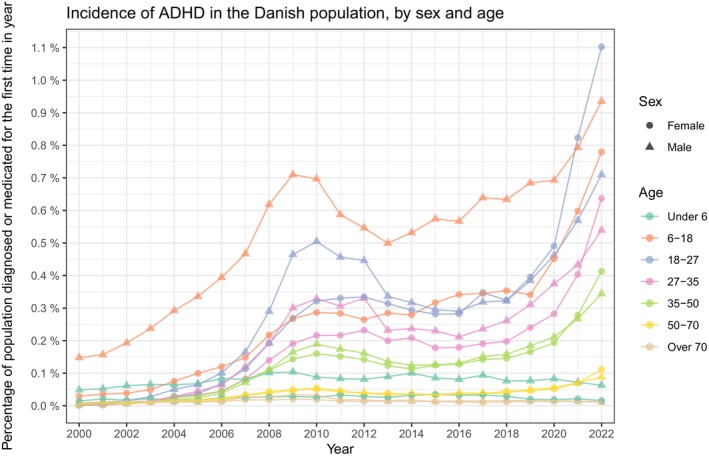
The incidence proportion of Attention Deficit/Hyperactivity Disorder (ADHD) in Denmark between the years 2000 and 2022. The entire population stratified by sex and age‐group.

For the age and sex stratified prevalence presented in Figure 3, the increase seen in the full population was present across all strata. Specifically, for the 18‐27‐year‐olds, the prevalence has increased over the past two decades to 8.16% (8.07%, 8.25%) for males and 6.12% (6.04%, 6.20%) for females in the year 2022. For the incidence, the same age group peaked within the females in 2022 at 1.10% (1.07%, 1.14%), whereas the male incidence was consistently highest in the 6–18 group, peaking in 2022 at 0.94% (0.91%, 0.97%). Age and sex stratified incidence proportions are presented in Figure 4. The number of prevalent and incident cases is found in Table S2. The age stratified male/female ratios of prevalence and incidence follow the overall decrease except for the youngest children aged under 6, which remain high throughout the study period (Figures S6, S7 and Table S2).

### 3.4 Medication

**FIGURE 5:**
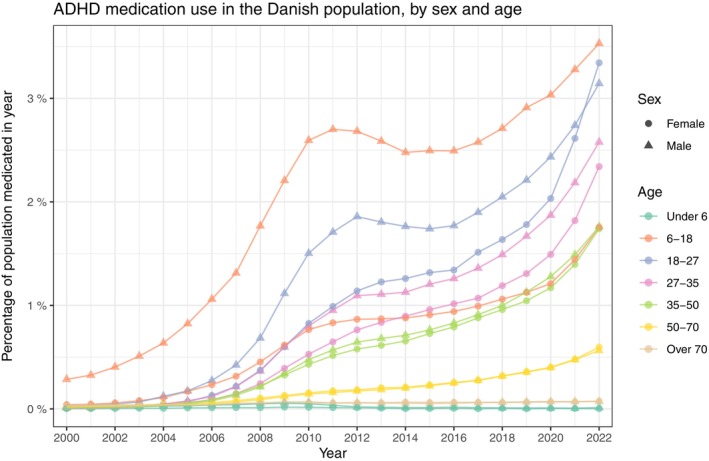
The use of medication for Attention Deficit/Hyperactivity Disorder (ADHD) in Denmark between the years 2000 and 2022. The entire population stratified by sex and age‐group.

**FIGURE 6:**
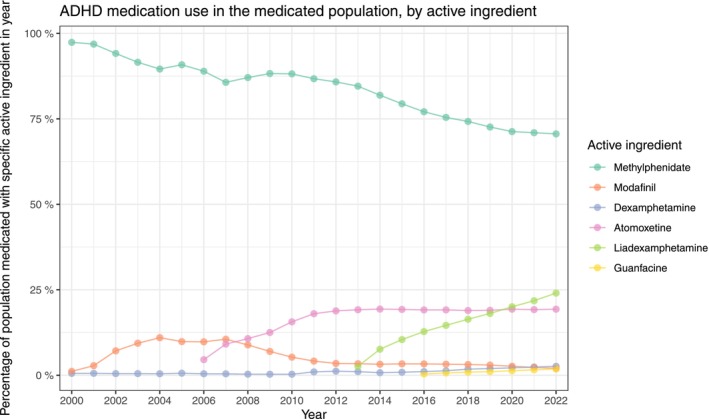
The use of medication for Attention Deficit/Hyperactivity Disorder (ADHD) in Denmark between the years 2000 and 2022. For all medicated patients, stratified by active ingredient in medication. Note that medications within a year does not necessarily sum to 100% as patients can redeem prescriptions for different medications within the same year.

The proportion of the Danish population who redeemed a prescription of ADHD medication in each year was increasing persistently throughout the study period for all age and sex strata. Young males were generally more medicated than young females (age groups 6–18 and 18–27), though 18‐27‐year‐old females, 3.34% (3.28%, 3.40%), surpassed males, 3.14% (3.08%, 3.20%), in 2022. The proportion of the population who redeemed medication is shown in Figure 5 and Table S2. The type of medication that was redeemed can be seen in Figure 6 and Table S1; here, it is evident that lisdexamphetamine was increasing in use from its introduction in 2013 to 24.03% (23.75%, 24.31%) in 2022, as atomoxetine was from its introduction in 2006 to 19.32% (19.06%, 19.59%) within the medicated population in 2022. Methylphenidate was the medication with the most redeemed prescriptions throughout the study period, though decreasing from being redeemed by 97.58% (96.91%, 98.25%) of the ADHD medicated patients in 2000 to 70.60% (70.29%, 70.90%) in 2022. The proportions of the Danish population redeeming prescriptions for each of the five types of medication were all increasing throughout the entire study period, as seen in Figure S3 and Table S1.

We conducted a supplementary analysis of age‐stratified prevalence and incidence proportions based only on hospital‐given diagnoses, thus excluding the medication‐driven classification of individuals in the population. In Figures S1, S2, and Table S3, we saw that the 2010 peak was substantially lower in young males.

## 4 Discussion

### 4.1 Key Results

For the entire Danish population, the prevalence of ADHD increased throughout the past two decades, reaching 3.03% (3.02%, 3.04%) in the year 2022. This increase is seen in the incidence as well, and most clearly for the 18–27‐year‐old females and females in general. Total medication use in the population was also increasing. Methylphenidate was the most commonly redeemed medication during the study period. However, in recent years, lisdexamphetamine has become increasingly popular. By 2022, one in four people using ADHD medication redeemed a prescription for lisdexamphetamine.

### 4.2 Strengths and Limitations

In Denmark, children and adolescents are usually diagnosed in a regional child and adolescent psychiatric department after being referred from the municipality‐level school system, whereas adults are most often diagnosed by private practice psychiatrists [30, 32]. The diagnoses given to children and adolescents were expected to be in The National Patient Register, whereas most adults were classified through The Danish National Prescription Registry. Combining both prescription data and hospital data, we expected to lower the extent of underestimation of the number of individuals with ADHD in Denmark. Due to the lack of information on diagnoses given by private practice psychiatrists, patients who were exclusively diagnosed and treated by private practice psychiatrists and did not redeem a prescription of ADHD medication were misclassified in this study.

To address potential biases in prevalence and incidence due to incomplete medical histories before 1994 and the adoption of ICD‐10 the same year, the study began in 2000, allowing a six‐year lead‐in period. Despite this, we expect that the prevalence and incidence are slightly under‐ and overestimated for the initial years of the study period. Categorizing based on ADHD medication could have biased the incidence in older adults upwards, as a study by Ormhøj et al. [33] in 2018 concluded that some ADHD medication was used off‐label as part of palliative treatment in older adults. While this might have been the case, a part of their argumentation was based on the inconsistency between ADHD diagnosis in the registers and medication use; however, most adults receive their diagnosis outside of the hospitals reporting to The National Patient Register, which may explain the majority of this discrepancy [32]. We further believe that the off‐label use of medication in palliative care would only have biased our estimates of the incidence, as the patients who received palliative care were not expected to live long enough to have affected the prevalence significantly. The study by Ormhøj et al. [33] was conducted on the years surrounding the peak in 2010 that we saw in our study. We believe that this temporary peak may be due to off‐label use, the subsequent National Health Authority bulletin sent out, and the medical community adhering here to [34, 35]. This off‐label use extends beyond the palliative use described by Ormhøj et al. [33] From the supplementary analysis of prevalences and incidences based only on hospital diagnoses, we observed that the incidence peak around 2010 was no longer recognizable among the elderly. This was most apparent when comparing the incidences in Figure 4 with those in Supplementary Figure 2. We concluded that Ormhøj et al. [33] might have been correct about off‐label use in the elderly, though it seemed much more present in the young males, presumably not reflecting palliative care.

### 4.3 Comparison With Current Literature

In 2015, Jensen et al. [36] showed that there was a rise in ADHD incidence rates from 1995 to 2010, with the expectation of further increase. Internationally, the prevalence and incidence have increased even further up until 2020 [37]. Using global spatial analysis on international meta‐data, the Danish prevalence in 2019 has previously been estimated to 0.7% [12, 13]. However, this estimate is largely underestimated compared to our register‐based analysis where the prevalence proportion in 2019 in Denmark was found to be 2.27% (2.25%, 2.28%).

We found similar results as Sørensen et al. [38] who studied ADHD medication use in Sweden, Norway, and Denmark from 2015 to 2020. They reported that 2.2% of the children and adolescents (age 5–19) in Denmark were using ADHD medicine in 2020. Our findings showed 3.03% (2.98%, 3.09%) for males and 1.21% (1.18%, 2.24%) for females in that year. They further found a significant increase in both the number of medication users and the amounts of medication consumed between 2015 and 2020 in all subgroups. In a broader perspective, Chan et al. [39] investigated the ADHD medication consumption in 64 countries and concluded that medication consumption was significantly increased across all countries with a health system comparable to the one in Denmark.

Kildegaard et al. [40] found similar results as we did for ADHD medication. They found an increase in depression medication, though not of the same magnitude as for ADHD, and little to no increase in the remaining psychiatric medications investigated. In the period from 2008 to 2018, there has been a 48% increase in the incidence of contact with psychiatric services among Danish children and adolescents (0–17 years old). In contrast, the incidence of schizophrenia was stable in Denmark, with no considerable increase over the past two decades [41, 42]. The steep rise we found in ADHD seems to be of a larger magnitude than the other psychiatric areas.

The underlying incidence in the population must have increased as well, as the 8.16% (8.07%, 8.25%) male and 6.12% (6.04%, 6.20%) female prevalences we observed in young Danish adults in 2022 are considerably higher than screening estimates from 2006, e.g. 4.4% lifetime prevalence in Americans [43], as well as more recent screening estimates being considerably higher as well, e.g. an ADHD prevalence of 15.5% in 2015 in American schoolchildren [16]. Referral for ADHD diagnosis requires that the symptoms significantly impair the individual. Therefore, there is a notable distinction between individuals formally diagnosed with ADHD and those who exhibit all the characteristics of ADHD but may not experience or report significant impairment [31]. Prevalence or incidence from an ADHD symptoms screening study, as the two American studies just mentioned, is therefore expected to be higher than the prevalence or incidence of diagnosed ADHD in a population. Unlike sample or screening studies, our methodology required that individuals meet the diagnostic criterion of experiencing enough impairment from their symptoms to seek psychiatric evaluation. While the prevalence of ADHD in Denmark has increased at a somewhat steady incline over time, our results show a more periodic increase in incidence with a decline from 2011 to 2014 and a steep increase around 2008 and again around 2021. These fluctuations may be explained by a means to settle accumulated waitlists as increased capacity demands are not timely handled. In Denmark, health care services are free and available for all citizens and usually handled in publicly owned and operated facilities. In 2019, The North Denmark Region made an agreement with a private psychiatric hospital to move some examination and initial treatment of patients with suspected ADHD or autism spectrum disorder to the private sector, showing an example of a sudden increase in capacity that could increase incidence temporarily. A concern in this case could be that the optimized and systematized ‘one size fits all’ examinations may have led to a narrow focus on the suspected disorder, disregarding the full picture of the patient and misdiagnosing the more complex patients.

Along with the increase we see for ADHD, there has been an increase in the general public psychiatric capacity, though not of the same magnitude [23, 44, 45]. The introduction of psychiatric pathway programs in 2017, which provided all public sectors with a systematized approach to early tracking and examination of children and adolescents with suspected ADHD, may have contributed to the increase in incidence. This could have had both direct and indirect effects by raising awareness of ADHD in the general population [46]. In addition, an evolving interpretation of ADHD diagnostic criteria may also have been a contributing factor to the increases in prevalence and incidence. Denmark used the ICD‐8 classification system until 1994, directly transitioning to ICD‐10 and skipping the ICD‐9 version. The ICD‐8 defined hyperkinetic symptoms narrowly, focusing primarily on severe manifestations of hyperactivity. In contrast, the ICD‐10, implemented in 1994, introduced a broader definition with the category of Hyperkinetic Disorders, encompassing codes F90.0, F90.1, and F90.8. This change expanded the diagnostic criteria to include a wider range of symptoms beyond severe hyperactivity, such as varying degrees of attention deficits, impulsivity, and less severe forms of hyperactivity. This broader scope has allowed clinicians to recognize and diagnose ADHD in a wider population, reflecting the contemporary understanding of the disorder as having varied manifestations. While these codes have been consistently used to diagnose ADHD throughout the study period, the clinical approach to diagnosing ADHD in Denmark may still have undergone changes over time. It is important to note that any references to the Diagnostic and Statistical Manual of Mental Disorders (DSM) in Danish clinical discussions are primarily for comparative or research purposes and do not reflect an adoption of DSM criteria in official diagnostic practices, which remain based on the ICD‐10 system. Regardless, clinical practice may have been influenced by an expanding international discourse on ADHD, and thus, have shifted clinical interpretation of ADHD [1, 30, 32, 47, 48, 49, 50].

### 4.4 Implications for Further Research and Policy

A large proportion of the steep increase in female ADHD prevalence, seen in the current study, could be attributed to a historical lack of awareness of ADHD in females. Previous studies on Scandinavian populations, including a Danish population, have found that the presence of ADHD‐related symptoms during adolescence is equally distributed between males and females [51, 52, 53]. This equal symptom burden suggests similar ADHD prevalence between sexes and an underdiagnosis of adolescent females. This is further supported by a Swedish study by Skoglund et al. which finds that females get diagnosed on average 3.9 years later than males, and that the proportion of ADHD patients who are still treated with ADHD medication five years after diagnosis is equal for women and men, indicating an equal need for treatment across sexes. In addition, the study by Skoglund et al. showed that females experience twice the psychiatric comorbidities as males [54].

Reliable forecasts and surveillance of the progress in ADHD incidence are warranted to allow timely adaptation of the health care system. As ADHD can have severe negative influence on individuals lives in society, research on adaptive measures such as reduction of risk factors and minimal cost family interventions is highly warranted.

## 5 Conclusion

Our study supports an increase in the prevalence of ADHD over the past two decades. This development has been further accelerated in the recent years 2020–2022 by an increase in the incidence. This increase in incidence is most pronounced amongst young females. Methylphenidate was consistently the most common ADHD drug prescribed, though lisdexamphetamine has been increasingly more prescribed over the past decade. The increase in prevalence may be attributed to changes in awareness and increased examination capacity as well as changes in the interpretation of diagnostic criteria inspired by clinical practice in other countries.

## Ethics Statement

Data was accessed through Statistics Denmark and handled in their environment in accordance with the Danish Data Protection Act. The project is registered at Aalborg University with the ID AAU031‐1022239.

## Conflicts of Interest

The authors declare no conflicts of interest.

### Peer Review

The peer review history for this article is available at https://www.webofscience.com/api/gateway/wos/peer‐review/10.1111/acps.13804.

## Supporting information


**Data S1** Figures.

**Supplementary Table S1.** Study time in days, as well as absolute numbers and proportion of prevalent, incident, and medicated patients with Attention Deficit Hyperactivity Disorder in the Danish population, stratified by year and sex with 95% confidence intervals. Each type of medication denoted by the corresponding Anatomical Therapeutic Chemical code.

**Supplementary Table S2.** Study time in days, as well as absolute numbers and proportion of prevalent, incident, and medicated patients with Attention Deficit Hyperactivity Disorder in the Danish population, stratified by year and sex. Male/female ratios by sex and age. All estimates with 95% confidence intervals.

**Supplementary Table S3.** Absolute numbers and proportion of prevalent and incident patients with a hospital given Attention Deficit Hyperactivity Disorder diagnosis in the Danish population, stratified by year and sex.

## Data Availability

Aggregated data used in this publication is published in the Supporting Information tables.

## References

[1] American Psychiatric Association, DSM‐5 Task Force , “Diagnostic and statistical manual of mental disorders: DSM‐5” 5th ed. (American Psychiatric Publishing, 2013), 10.1176/appi.books.9780890425596.

[2] N. Kotsopoulos , M. P. Connolly , E. Sobanski , and M. J. Postma , “Assessing the Economic Burden and Benefit–Cost of Treating Attention‐Deficit Hyperactivity Disorder in Germany,” Journal of Health Economics and Outcomes Research 1, no. 3 (2013): 212–223, 10.36469/9866. 37662879 PMC10471420

[3] E. Anker , A. Halmøy , and T. Heir , “Work Participation in ADHD and Associations With Social Characteristics, Education, Lifetime Depression, and ADHD Symptom Severity,” ADHD Attention Deficit and Hyperactivity Disorders 11, no. 2 (2019): 159–165, 10.1007/s12402-018-0260-2. 30187383

[4] M. L. Danielson , J. R. Holbrook , R. H. Bitsko , et al., “State‐Level Estimates of the Prevalence of Parent‐Reported ADHD Diagnosis and Treatment Among U.S. Children and Adolescents, 2016 to 2019,” Journal of Attention Disorders 26, no. 13 (2022): 1685–1697, 10.1177/10870547221099961. 35603751 PMC9489617

[5] T. Li , B. Franke , A. AriasVasquez , and N. R. Mota , “Mapping Relationships Between ADHD Genetic Liability, Stressful Life Events, and ADHD Symptoms in Healthy Adults,” American Journal of Medical Genetics, Part B: Neuropsychiatric Genetics 186, no. 4 (2021): 242–250, 10.1002/ajmg.b.32828. PMC8359274 33319511

[6] J. C. Agnew‐Blais , G. V. Polanczyk , A. Danese , J. Wertz , T. E. Moffitt , and L. Arseneault , “Evaluation of the Persistence, Remission, and Emergence of Attention‐Deficit/Hyperactivity Disorder in Young Adulthood,” JAMA Psychiatry 73, no. 7 (2016): 713, 10.1001/jamapsychiatry.2016.0465. 27192174 PMC5475268

[7] L. Hakkaart‐van Roijen , B. W. C. Zwirs , C. Bouwmans , et al., “Societal Costs and Quality of Life of Children Suffering From Attention Deficient Hyperactivity Disorder (ADHD),” European Child & Adolescent Psychiatry 16, no. 5 (2007): 316–326, 10.1007/s00787-007-0603-6. 17483870

[8] A. R. Altszuler , T. F. Page , E. M. Gnagy , et al., “Financial Dependence of Young Adults With Childhood ADHD,” Journal of Abnormal Child Psychology 44, no. 6 (2016): 1217–1229, 10.1007/s10802-015-0093-9. 26542688 PMC4887412

[9] M. A. Ramos Olazagasti , R. G. Klein , S. Mannuzza , et al., “Does Childhood Attention‐Deficit/Hyperactivity Disorder Predict Risk‐Taking and Medical Illnesses in Adulthood?,” Journal of the American Academy of Child and Adolescent Psychiatry 52, no. 2 (2013): 153–162.e4, 10.1016/j.jaac.2012.11.012. 23357442 PMC3662801

[10] J. E. Max , K. Mathews , F. F. Manes , et al., “Attention Deficit Hyperactivity Disorder and Neurocognitive Correlates After Childhood Stroke,” Journal of the International Neuropsychological Society 9, no. 6 (2003): 815–829, 10.1017/S1355617703960012. 14632240

[11] D. E. Babinski , J. R. Mazzant , B. M. Merrill , et al., “Lifetime Caregiver Strain Among Mothers of Adolescents and Young Adults With Attention‐Deficit/Hyperactivity Disorder,” Journal of Family Psychology 34, no. 3 (2020): 342–352, 10.1037/fam0000609. 31750692 PMC7102920

[12] Global, Regional, and National Burden of 12 Mental Disorders in 204 Countries and Territories, 1990–2019: A Systematic Analysis for the Global Burden of Disease Study 2019. Lancet Psychiatry 9, no. 2 (2022): 137–150, 10.1016/S2215-0366(21)00395-3. 35026139 PMC8776563

[13] S. Cortese , M. Song , L. C. Farhat , et al., “Incidence, Prevalence, and Global Burden of ADHD From 1990 to 2019 Across 204 Countries: Data, With Critical Re‐Analysis, From the Global Burden of Disease Study,” Molecular Psychiatry 28, no. 11 (2023): 4823–4830, 10.1038/s41380-023-02228-3. 37684322

[14] G. Ayano , S. Demelash , Y. Gizachew , L. Tsegay , and R. Alati , “The Global Prevalence of Attention Deficit Hyperactivity Disorder in Children and Adolescents: An Umbrella Review of Meta‐Analyses,” Journal of Affective Disorders 339 (2023): 860–866, 10.1016/J.JAD.2023.07.071. 37495084

[15] G. Ayano , L. Tsegay , Y. Gizachew , et al., “Prevalence of Attention Deficit Hyperactivity Disorder in Adults: Umbrella Review of Evidence Generated Across the Globe,” Psychiatry Research 328 (2023): 115449, 10.1016/J.PSYCHRES.2023.115449. 37708807

[16] A. S. Rowland , B. J. Skipper , D. M. Umbach , et al., “The Prevalence of ADHD in a Population‐Based Sample,” Journal of Attention Disorders 19, no. 9 (2015): 741–754, 10.1177/1087054713513799. 24336124 PMC4058092

[17] M. Polyzoi , E. Ahnemark , E. Medin , and Y. Ginsberg , “Estimated Prevalence and Incidence of Diagnosed ADHD and Health Care Utilization in Adults in Sweden—A Longitudinal Population‐Based Register Study,” Neuropsychiatric Disease and Treatment 14 (2018): 1149–1161, 10.2147/NDT.S155838. 29765219 PMC5944447

[18] S. E. Holden , S. Jenkins‐Jones , C. D. Poole , C. L. Morgan , D. Coghill , and C. J. Currie , “The Prevalence and Incidence, Resource Use and Financial Costs of Treating People With Attention Deficit/Hyperactivity Disorder (ADHD) in the United Kingdom (1998 to 2010),” Child and Adolescent Psychiatry and Mental Health 7, no. 1 (2013): 1–13, 10.1186/1753-2000-7-34/TABLES/4. 24119376 PMC3856565

[19] S. Grøntved , M. Jørgine Kirkeby , S. Paaske Johnsen , J. Mainz , J. Brink Valentin , and J. C. Mohr , “Towards Reliable Forecasting of Healthcare Capacity Needs: A Scoping Review and Evidence Mapping,” International Journal of Medical Informatics 189 (2024): 105527, 10.1016/J.IJMEDINF.2024.105527. 38901268

[20] Lægemøde , “styrk psykiatrien nu. *Styrk Psykiatrien Nu—Det Vedrører Os Alle* ,” 2018.

[21] Dansk Psykiatrisk Selskab , Dansk Psykiatrisk Selskabs Hvidbog 2021–2031 (Dansk Psykiatrisk Selskab, 2021), https://dpsnet.dk/wp‐content/uploads/2021/10/DPS_hvidbog_2021‐2031.pdf.

[22] E. Hewlett and V. Moran , Making Mental Health Count: The Social and Economic Costs of Neglecting Mental Health Care (OECD Health Policy Studies, 2014;(July)), 10.1787/9789264208445-en.

[23] Danske Regioner , “ Analyse Af Psykiatriområdet ,” 2020, accessed August 22, 2024, https://bedrepsykiatri.dk/wp‐content/uploads/2020/09/2020‐Regioner‐analyse‐af‐psykiatriomraadet‐danske‐regioner‐september‐2020.pdf.

[24] J. P. Vandenbroucke , E. Von Elm , D. G. Altman , et al., “Strengthening the Reporting of Observational Studies in Epidemiology (STROBE): Explanation and Elaboration,” Epidemiology 18, no. 6 (2007): 805–835, 10.1097/EDE.0B013E3181577511. 18049195

[25] M. Schmidt , S. A. J. Schmidt , K. Adelborg , et al., “The Danish Health Care System and Epidemiological Research: From Health Care Contacts to Database Records,” Clinical Epidemiology 11, no. 11 (2019): 563–591, 10.2147/CLEP.S179083. 31372058 PMC6634267

[26] M. Schmidt , S. A. J. Schmidt , J. L. Sandegaard , V. Ehrenstein , L. Pedersen , and H. T. Sørensen , “The Danish National Patient Registry: A Review of Content, Data Quality, and Research Potential,” Clinical Epidemiology 7 (2015): 449–490, 10.2147/CLEP.S91125. 26604824 PMC4655913

[27] H. W. Kildemoes , H. T. Sørensen , and J. Hallas , “The Danish National Prescription Registry,” Scandinavian Journal of Public Health 39, no. 7_suppl (2011): 38–41, 10.1177/1403494810394717. 21775349

[28] J. Mainz , M. H. Hess , and S. P. Johnsen , “The Danish Unique Personal Identifier and the Danish Civil Registration System as a Tool for Research and Quality Improvement,” International Journal for Quality in Health Care 31, no. 9 (2019): mzz008, 10.1093/intqhc/mzz008. 31220255

[29] K. Helweg‐Larsen , “The Danish Register of Causes of Death,” Scandinavian Journal of Public Health 39, no. 7_suppl (2011): 26–29, 10.1177/1403494811399958. 21775346

[30] Sundhedsstyrrelsen , “ National Klinisk Retningslinje for Udredning Og Behandling Af ADHD Hos Børn Og Unge ,” 2018, accessed January 23, 2025, https://www.sst.dk/‐/media/Udgivelser/2018/National‐Klinisk‐Retningslinje‐Udredning‐og‐behandling‐af‐ADHD‐hos‐boern‐og‐unge.

[31] National Collaborating Centre for Mental Health , Attention Deficit Hyperactivity Disorder: Diagnosis and Management. Nice Guideline n^o^ 87 (National Institute for Health and Clinical Excellence, 2018;(September 2019)), 62, www.nice.org.uk/guidance/ng87. 29634174

[32] Sundhedsstyrrelsen , “ National Klinisk Retningslinje for Udredning Og Behandling Af ADHD Hos Voksne—Med Forstyrrelser Af Aktivitet Og Opmærksomhed Samt Opmærksomhedsforstyrrelse Uden Hyperaktivitet ,” 2015, accessed January 23, 2025, https://www.sst.dk/da/nyheder/2015/~/media/91742BB2537F480E9481E415144C3687.ashx.

[33] S. S. Ormhøj , A. Pottegård , C. Gasse , and L. Rasmussen , “Use of Attention‐Deficit/Hyperactivity Disorder Medication Among Older Adults in Denmark,” British Journal of Clinical Pharmacology 84, no. 7 (2018): 1505–1513, 10.1111/BCP.13569. 29493809 PMC6005604

[34] Lægemiddelstyrelsen , “ Fokusrapport, Vurdering Af Sikkerheden Ved Brug Af Methylphenidat ,” 2010.

[35] Ritalin og andre centralstimulerende lægemidler skal opbevares forsvarligt, og kun tages af patienter | Ugeskriftet.dk accessed June 2, 2024, https://ugeskriftet.dk/nyhed/ritalin‐og‐andre‐centralstimulerende‐laegemidler‐skal‐opbevares‐forsvarligt‐og‐kun‐tages‐af.

[36] C. M. Jensen and H. C. Steinhausen , “Time Trends in Incidence Rates of Diagnosed Attention‐Deficit/Hyperactivity Disorder Across 16 Years in a Nationwide Danish Registry Study,” Journal of Clinical Psychiatry 76, no. 03 (2015): e334–e341, 10.4088/JCP.14m09094. 25830455

[37] L. Kazda , K. Bell , R. Thomas , K. McGeechan , R. Sims , and A. Barratt , “Overdiagnosis of Attention‐Deficit/Hyperactivity Disorder in Children and Adolescents: A Systematic Scoping Review,” JAMA Network Open 4, no. 4 (2021): e215335, 10.1001/JAMANETWORKOPEN.2021.5335. 33843998 PMC8042533

[38] A. M. S. Sørensen , R. Wesselhöeft , J. H. Andersen , et al., “Trends in Use of Attention Deficit Hyperactivity Disorder Medication Among Children and Adolescents in Scandinavia in 2010–2020,” European Child & Adolescent Psychiatry 32, no. 10 (2023): 2049–2056, 10.1007/s00787-022-02034-2. 35831669

[39] A. Y. L. Chan , T. T. Ma , W. C. Y. Lau , et al., “Attention‐Deficit/Hyperactivity Disorder Medication Consumption in 64 Countries and Regions From 2015 to 2019: A Longitudinal Study,” EClinicalMedicine 58 (2023): 101780, 10.1016/j.eclinm.2022.101780. 37181411 PMC10166776

[40] H. Kildegaard , R. Wesselhoeft , L. C. Lund , and M. Bliddal , “Post‐Pandemic Trends in Psychotropic Medication Use in Danish Children, Adolescents, and Young Adults,” Acta Psychiatrica Scandinavica 150, no. 3 (2024): 174–177, 10.1111/acps.13719. 38881268

[41] Indenrigs‐ og Boligmiisteriets Benchmarkingenhed , “ Børn Og Unge Med Psykiatrisk Debut ,” 2021, accessed August 22, 2024, https://www.benchmark.dk/Media/637550392299995206/Hovedrapport%20‐%20B%c3%b8rn%20og%20unge%20med%20psykiatrisk%20debut.pdf.

[42] O. Köhler‐Forsberg , S. Antonsen , C. B. Pedersen , P. B. Mortensen , J. J. McGrath , and O. Mors , “Schizophrenia Spectrum Disorders in Denmark Between 2000 and 2018: Incidence and Early Diagnostic Transition,” Acta Psychiatrica Scandinavica 148, no. 2 (2023): 190–198, 10.1111/acps.13565. 37237326

[43] R. C. Kessler , L. Adler , R. Berkley , et al., “The Prevalence and Correlates of Adult ADHD in the United States: Results From the National Comorbidity Survey Replication,” American Journal of Psychiatry 163, no. 4 (2006): 716–723, 10.1176/ajp.2006.163.4.716. 16585449 PMC2859678

[44] Psykiatrien i Region Nordjylland , Tilkøb af udrednings‐ og behandlingskapacitet i Børne‐ og Ungdomspsykiatrisk Afdeling accessed August 22, 2024, https://psykiatri.rn.dk/da/For‐sundhedsfaglige/Rammer‐og‐aftaler/Tilkoeb‐af‐udrednings‐og‐behandlingskapacitet‐i‐Boerne‐og‐Ungdomspsykiatrisk‐Afdeling.

[45] Sundhedsstyrrelsen , “ Specialevejledning for Børne‐ Og Ungdomspsykiatri ,” 2021, accessed August 22, 2024, https://www.sst.dk/‐/media/Viden/Specialplaner/Specialeplan‐for‐b%C3%B8rne‐‐og‐ungdomspsykiatri/Specialevejledning‐Boerne‐‐og‐ungdomspsykiatri‐220621.ashx.

[46] Sundhedsstyrrelsen , “ Førløbsprogram for Børn Og Unge Med ADHD ,” 2017, accessed August 22, 2024, https://www.sst.dk/‐/media/Udgivelser/2017/Forloebsprogrammer/Forl%C3%B8bsprogram‐for‐b%C3%B8rn‐og‐unge‐med‐ADHD.ashx.

[47] Sundhedsstyrelsen , “National Klinisk Retningslinje for Udredning Og Behandling Af ADHD Hos Børn Og Unge—Med Fokus På Diagnoserne” Forstyrrelse Af Aktivitet Og Opmærksomhed” Og” Opmærksomhedsforstyrrelse Uden Hyperaktivitet” i Henhold Til ICD‐10,” 2014.

[48] Sundhedsstyrrelsen , “ National Klinisk Retningslinje for Udredning Og Behandling Af ADHD Hos Voksne ,” 2017, accessed January 23, 2025, https://adhd.dk/wp‐content/uploads/2022/12/National‐Klinisk‐Retningslinje‐ADHD‐hos‐voksne.pdf.

[49] Sundhedsstyrrelsen , “ National Klinisk Retningslinje for Udredning Og Behandling Af ADHD Hos Børn Og Unge ,” 2021, accessed January 23, 2025, https://www.sst.dk/‐/media/Udgivelser/2021/NKR‐ADHD‐boern‐og‐unge/NKR‐udredning‐og‐behandling‐af‐ADHD‐hos‐boern‐og‐unge.ashx.

[50] American Psychiatric Association , Diagnostic and Statistical Manual of Mental Disorders, 4th ed. (American Psychiatric Publishing, Inc., 1994), https://psycnet.apa.org/record/1994‐97698‐000.

[51] B. Van Roy , B. Grøholt , S. Heyerdahl , and J. Clench‐Aas , “Self‐Reported Strengths and Difficulties in a Large Norwegian Population 10–19 Years: Age and Gender Specific Results of the Extended SDQ‐Questionnaire,” European Child & Adolescent Psychiatry 15, no. 4 (2006): 189–198, 10.1007/s00787-005-0521-4. 16724172

[52] L. G. Lundh , M. Wangby‐Lundh , and J. Bjärehed , “Self‐Reported Emotional and Behavioral Problems in Swedish 14 to 15‐Year‐Old Adolescents: A Study With the Self‐Report Version of the Strengths and Difficulties Questionnaire,” Scandinavian Journal of Psychology 49, no. 6 (2008): 523–532, 10.1111/j.1467-9450.2008.00668.x. 18489532

[53] P. Due , F. Diderichsen , C. Meilstrup , et al., “Børn og Unges Mentale Helbred: Forekomst af psykiske symptomer og lidelser og mulige forebyggelsesindsatser,” 2014.

[54] C. Skoglund , I. Sundström Poromaa , D. Leksell , et al., “Time After Time: Failure to Identify and Support Females With ADHD—A Swedish Population Register Study,” Journal of Child Psychology and Psychiatry 65, no. 6 (2024): 832–844, 10.1111/jcpp.13920. 38016697

