# Supplementary material for: Prevalence and Incidence of Attention Deficit/Hyperactivity Disorder in Denmark. A National Register‐Based Open Cohort Study
Source: Acta Psychiatr Scand. 2025 Mar 27;152(1):27–38. doi: 10.1111/acps.13804 (PMC12127063; doi:10.1111/acps.13804)
Supplement: Supplementary file 1 — Data S1 Figures. [file ACPS-152-27-s002.docx]

**Supplementary materials for “**Prevalence and Incidence of Attention Deficit/Hyperactivity Disorder in Denmark. A national register-based open cohort study.”

Data

Three excel files with the data for the plots, as well 95% confidence intervals for the presented estimates are appended as “Supplementary Table 1”, “Supplementary Table 2”, and “Supplementary Table 3”

Supplementary figures


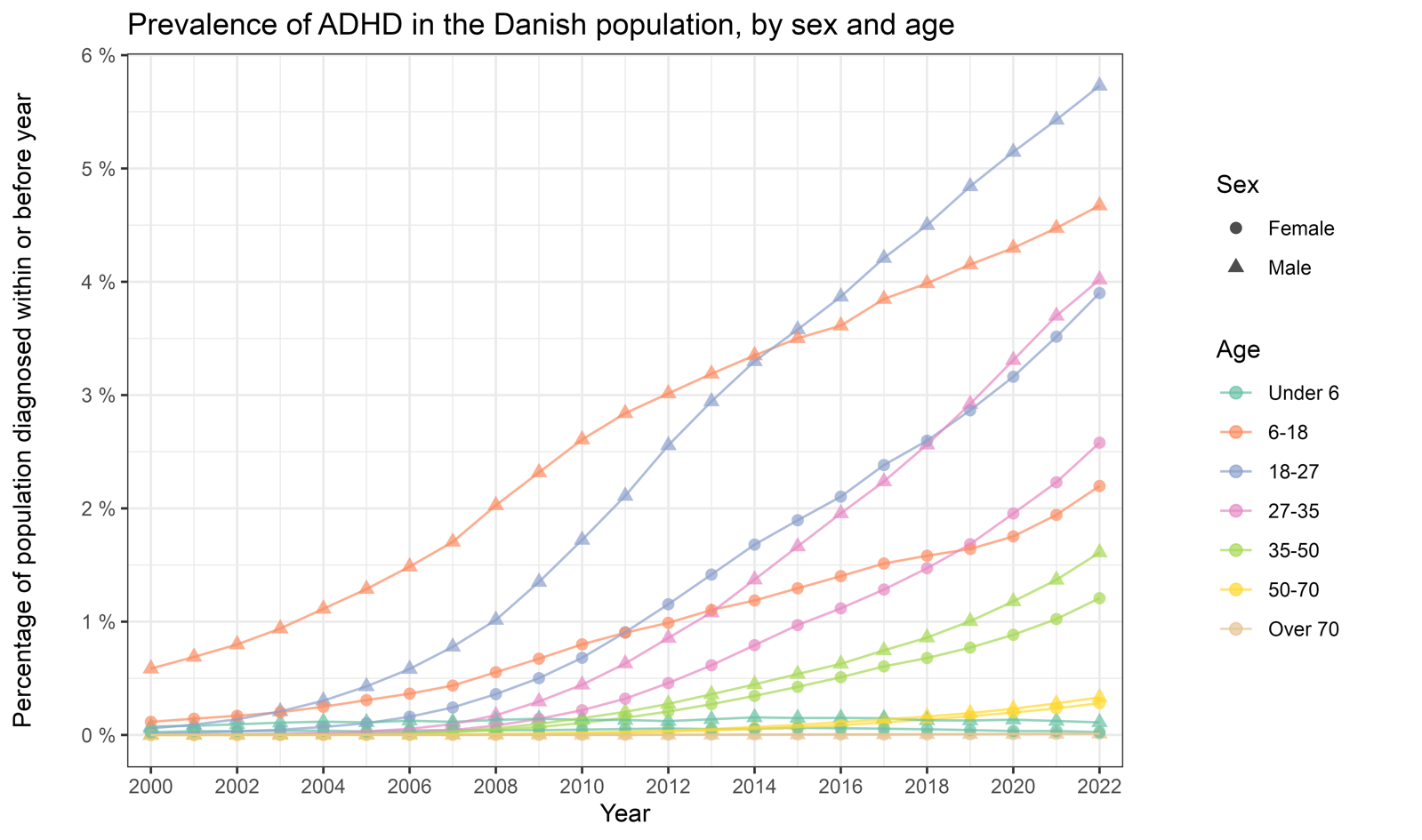


**Supplementary Figure 1:** *The prevalence proportion of Attention Deficit/Hyperactivity Disorder (ADHD) in Denmark between the years 2000 and 2022. The entire population stratified by sex and age-group. Only based on hospital recorded ADHD, thus not categorizing on medication use.*


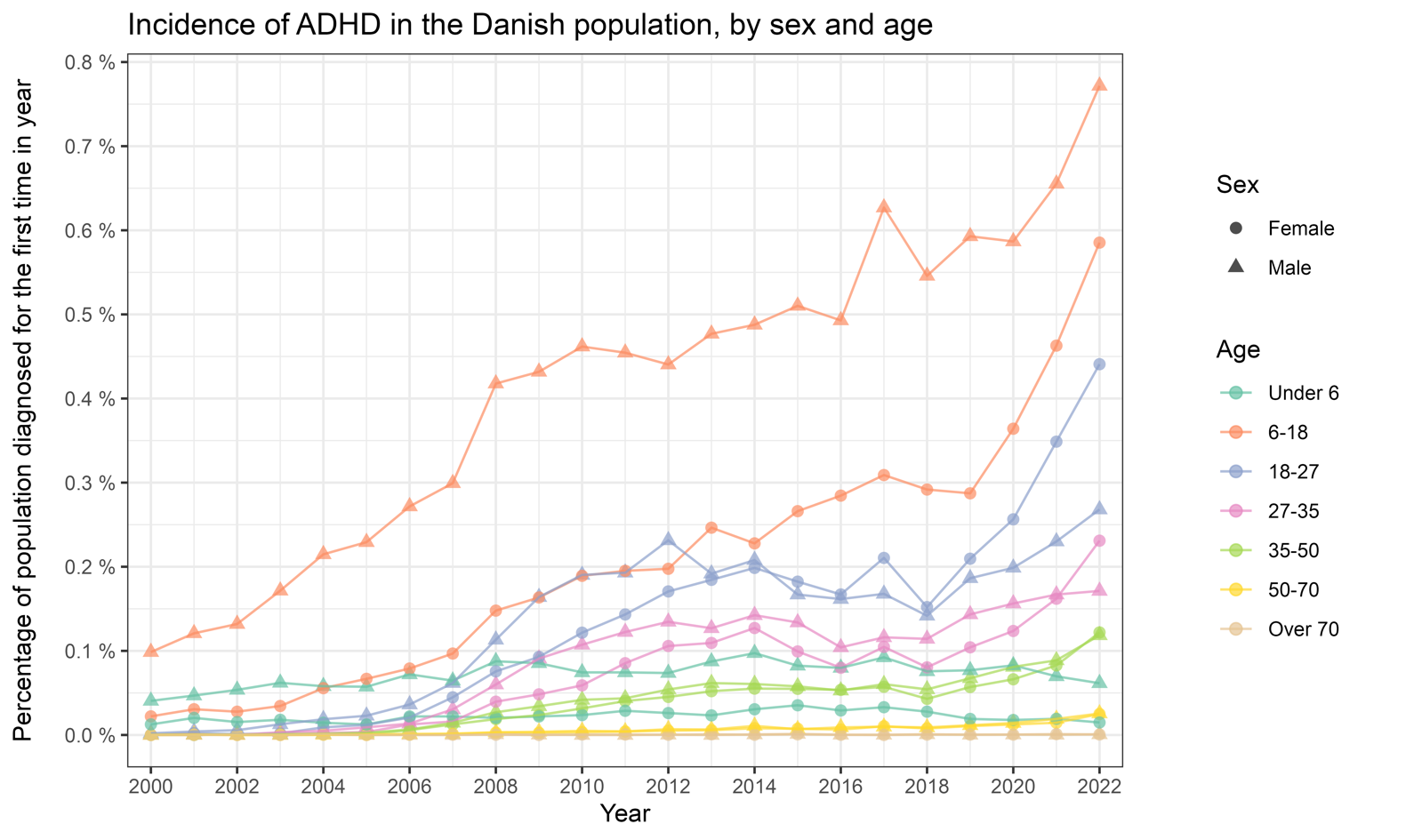


**Supplementary Figure 2:** *The incidence proportion of Attention Deficit/Hyperactivity Disorder (ADHD) in Denmark between the years 2000 and 2022. The entire population stratified by sex and age-group. Only based on hospital recorded ADHD, thus not categorizing on medication use.*


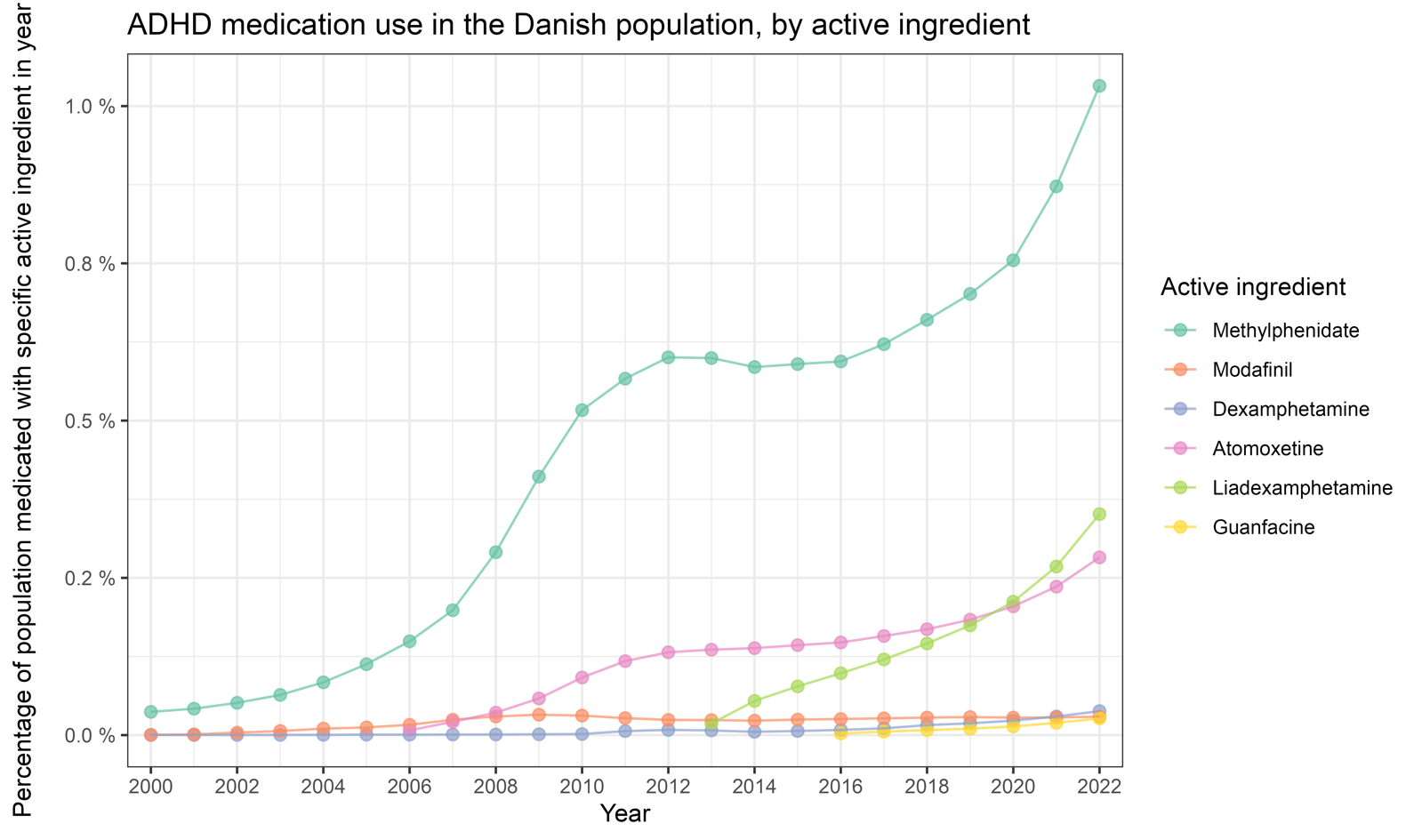


**Supplementary Figure 3:** *The use of medication for Attention Deficit/Hyperactivity Disorder (ADHD) in Denmark between the years 2000 and 2022. The entire population stratified by active ingredient in medication.*


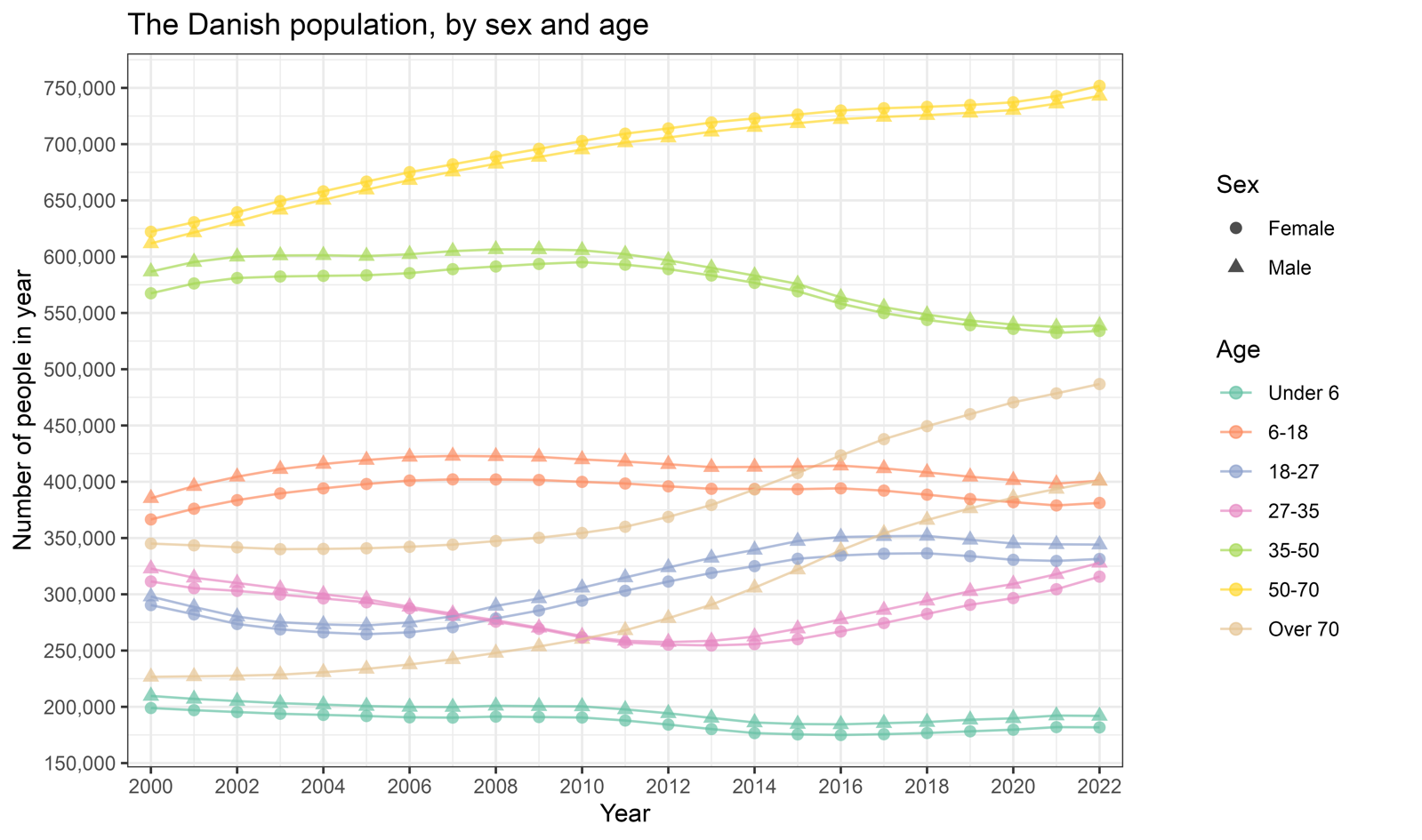


**Supplementary Figure 4:** *The numeric population living in Denmark a given year between the years 2000 and 2022. Stratified by sex and age-group.*


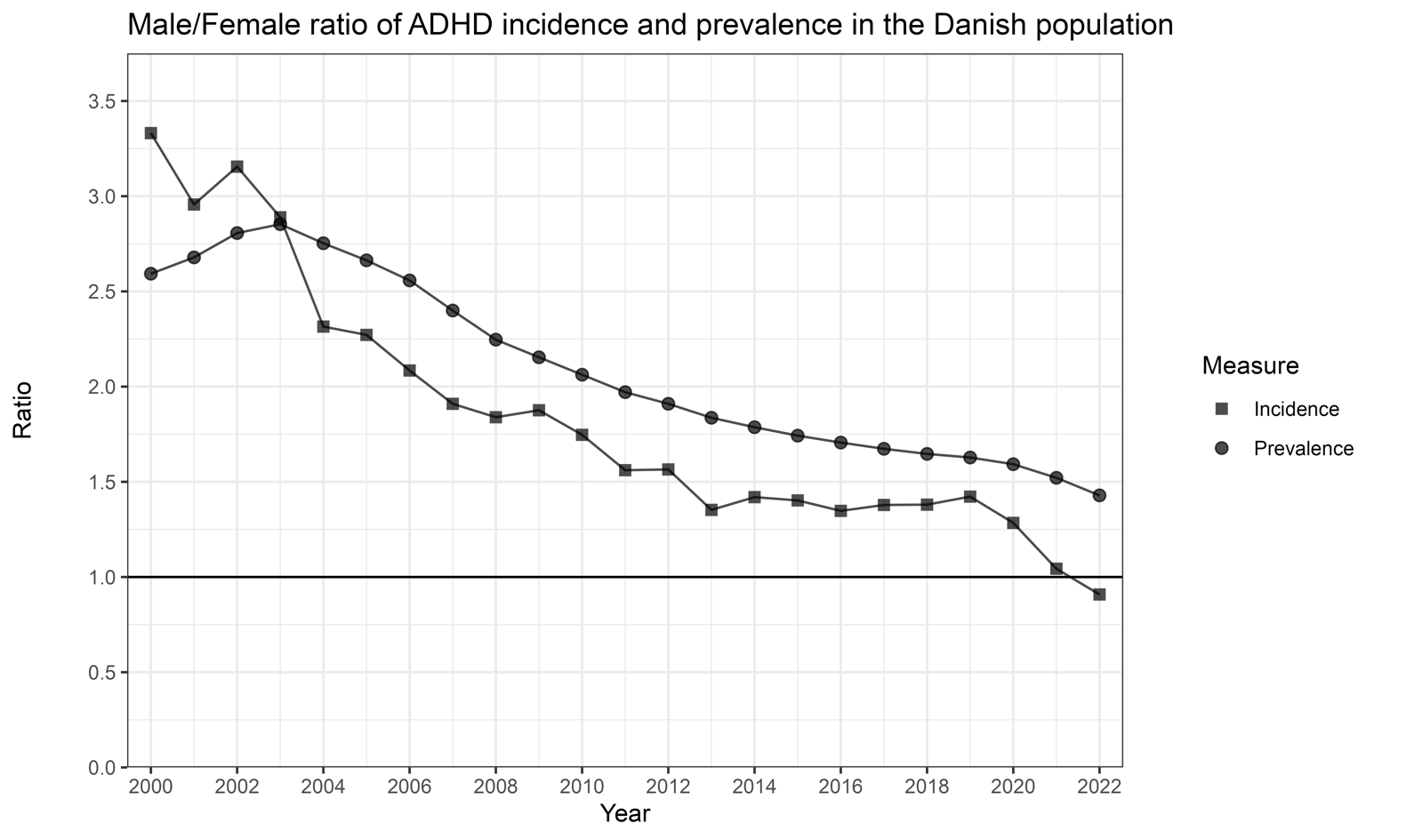


**Supplementary Figure 5:** *The ratio between the male and female ADHD-prevalence, and the ratio between the male and female ADHD-incidence. In Denmark a given year between the years 2000 and 2022.*


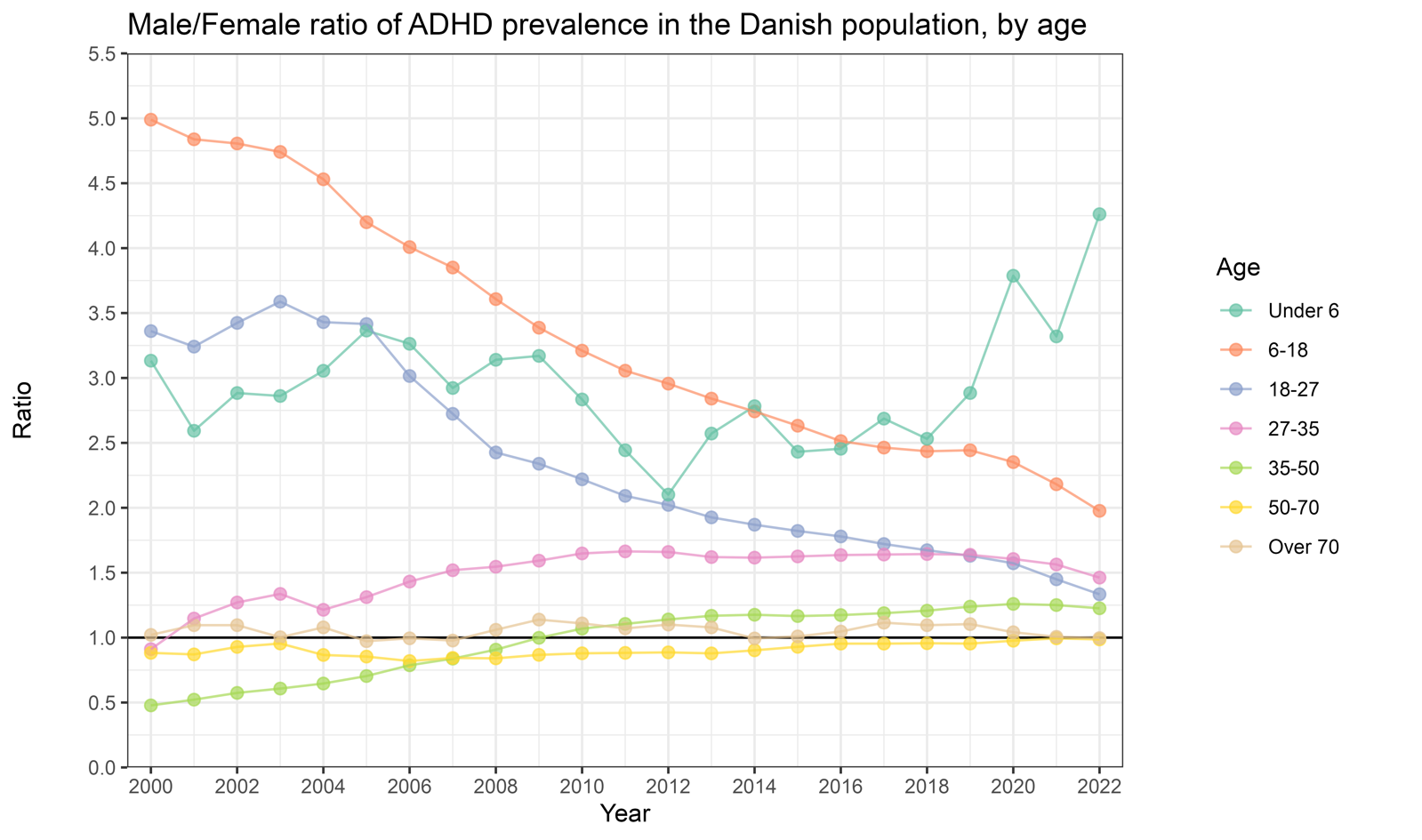


**Supplementary Figure 6:** *The ratio between the male and female ADHD-prevalence. In Denmark a given year between the years 2000 and 2022. Stratified by age-group.*


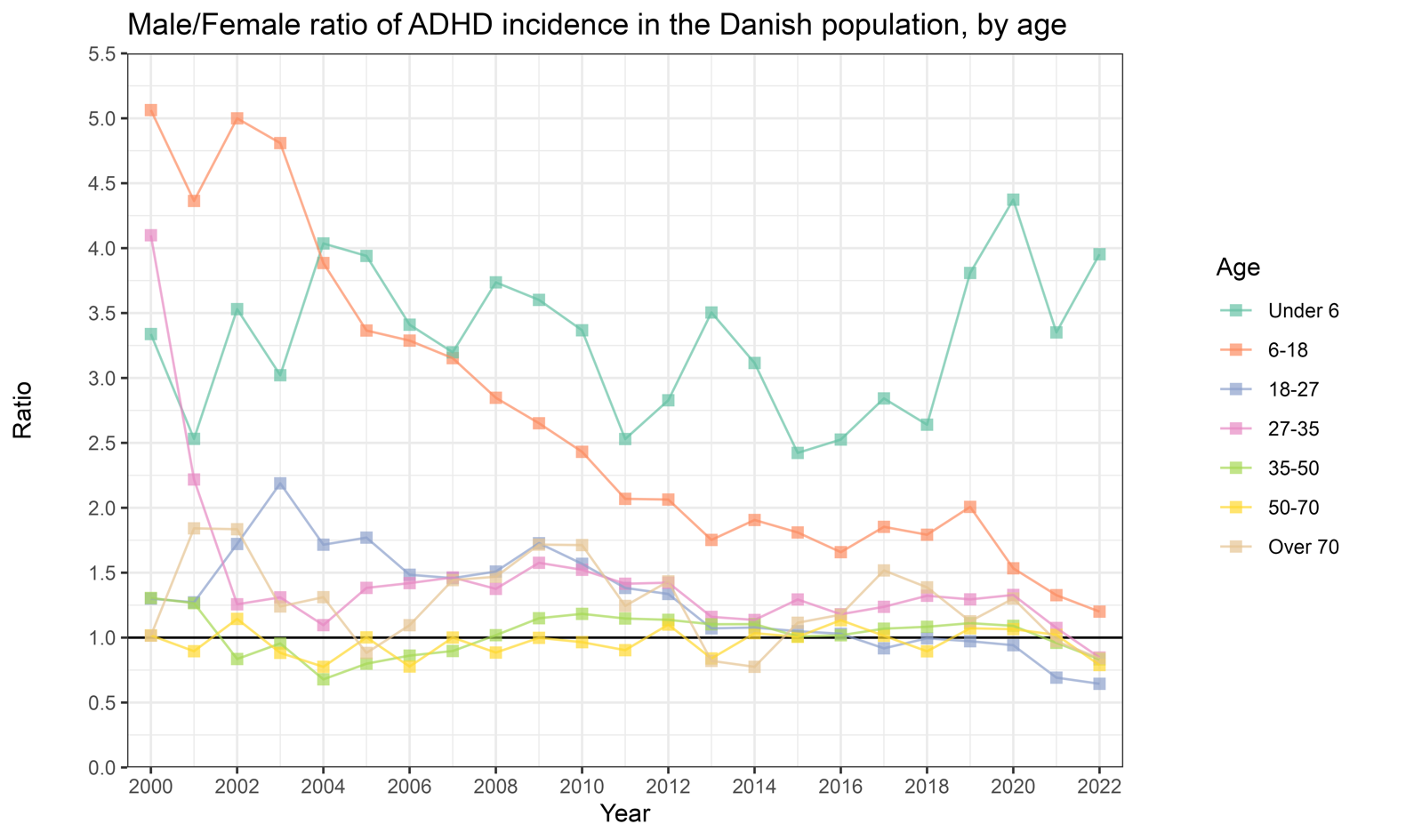


**Supplementary Figure 7:** *The ratio between the male and female ADHD-incidence. In Denmark a given year between the years 2000 and 2022. Stratified by age-group.*
